# Performance analysis of PPP models in rural tourism projects of Shandong Province based on DEA and super-DEA

**DOI:** 10.1371/journal.pone.0312380

**Published:** 2024-12-05

**Authors:** Ruimin Dai, Changli Zhang

**Affiliations:** 1 School of Public Policy & Management, China University of Mining and Technology, Xuzhou, China; 2 School of Modern Service Management, Shandong Youth University of Political Science, Jinan, China; Pablo de Olavide University: Universidad Pablo de Olavide, SPAIN

## Abstract

Rural tourism development has made positive contributions to promoting an increase in farmers’ income, coordinated urban-rural development, rural civilization, and industrial transformation & upgrading. However, it also faces problems such as immature development and unsound planning. This paper focuses on the development status of the public-private partnership (PPP) models in rural tourism projects of Shandong Province in China, as well as their operations and cooperation models. Then, this paper assesses the impacts of PPP models on the rural tourism industry of Shandong Province, and appraises their performance by combining standard data envelopment analysis (DEA) and super-DEA models. The main findings are (1) The overall efficiency of PPP models in rural tourism projects of Shandong Province is moderate, and resource utilization is disproportionate to output, leaving great room for improvement. (2) PPP models in Larger projects with more complexity, are more easily tending to a lower overall efficiency. (3) The optimal development PPP model for rural tourism projects of Shandong Province is “Stock Project + Build-Operate-Transfer (BOT) model”. In response to these findings, this paper gives some targeted opinions and suggestions, including strengthening government supervision, controlling project scale, incentivizing enterprises to play intermediary roles, and innovating PPP models. In this way, it provides strong academic support and useful policy suggestions for the sustainable development of PPP models in worldwide rural tourism.

## Introduction

In recent years, with the substantial support of national preferential policies, rural revitalization strategies, and relevant government departments, rural tourism projects with rich local features and ethnic cultural customs have sprung up all across China. According to the 2022 White Paper on Rural Revitalization and Development issued by Ctrip, the largest online travel agency in China, the number of rural homestays registered on Ctrip alone has increased from around 70,000 in 2017 to over 300,000 in 2022, with an average annual growth rate above 60%. This increase testifies the popularity of the rural tourism market(Above date sources: https://finance.sina.com.cn/jjxw/2023-01-10/doc-imxzrxks8608062.shtml). At the same time, the rapid development of rural tourism has also driven the significant progress in tourism infrastructure, scale of the tourism industry, tourism environmental sanitation, etc. Rural tourism has made positive contributions to promoting an increase in farmers’ income, coordinated urban-rural development, rural civilization, and industrial transformation & upgrading. Such as, in Shandong Province, located in east coast of China with developed economic and rural tourism market, the added value of agriculture has recently exceeded 1.2 times and the per capita income of farmers has increased by 1,950 CNY, benefiting from the development of rural tourism(Above date sources: https://www.dzwww.com/shandong/sdnews/201706/t20170614_16043465.htm).

However, due to the high competitiveness of the rural tourism market, achieveing continuous development, operations, and innovation are becoming essential for rural tourism projects. While their small scale and long payback characterics also make it difficult for them to attract the attention or investment of large financial institutions. Thus, investors of rural tourism projects in practice are mostly farmers and village collectives with limited funding sources, which are often unable to meet the capital demand of rural tourism projects construction and operation. Consequencely, limited by funding, technology and management, some rural tourism projects are inadequate in terms of infrastructure construction, such as transportation, water, electricity, and communication, which also constrain the development of rural tourism.

Public-private partnership (PPP) refers to an efficient financing model in which the government and social capital (including enterprises, institutions, and individuals) sign cooperation agreements to share risks, revenues, management and power in investment, construction, operation, and maintenance of public projects. So far, PPP models have been widely used in national infrastructure construction and grassroots management, their applications in the tourism field are also particularly broad in China. The country has also become aware of the enormous potential of PPP models in tourism development, as well as their alignment with the concepts of promoting culture-tourism integration and rural tourism development under the rural revitalization strategies. The first rural focused policy statement released every year by Chinese Central Government, has explicitly proposed to guide and support social capital to develop leisure tourism projects with high involvement of farmers and a wide range of benefits in 2017, affirming the combination of PPP and rural leisure tourism policy. Applying PPP models to the development and construction of rural tourism, can meet the capital demand of rural tourism, alleviate the financial pressure on the government, and open up new areas for social capital investment. In Oct. 2018, 13 departments and commissions including the National Development and Reform Commission and the Ministry of Culture and Tourism of China, jointly issued the Action Plan on Improving and Upgrading Rural Tourism (2018–2020), which encourages social capital and private investment to participate in the development and construction of rural tourism, and guides private captial to take part in rural infrastructure and tourism projects yielding certain revenues through PPP models or establishments with public funding then transfering for private operations. Undoubtedly, PPP models have provided a trustworthy path for rural tourism projects construction addressing the issue of insufficient funds, and enhanced benefits of rural tourism development in income increase, rural civilization and industrial upgrading.

**Fig 1:**
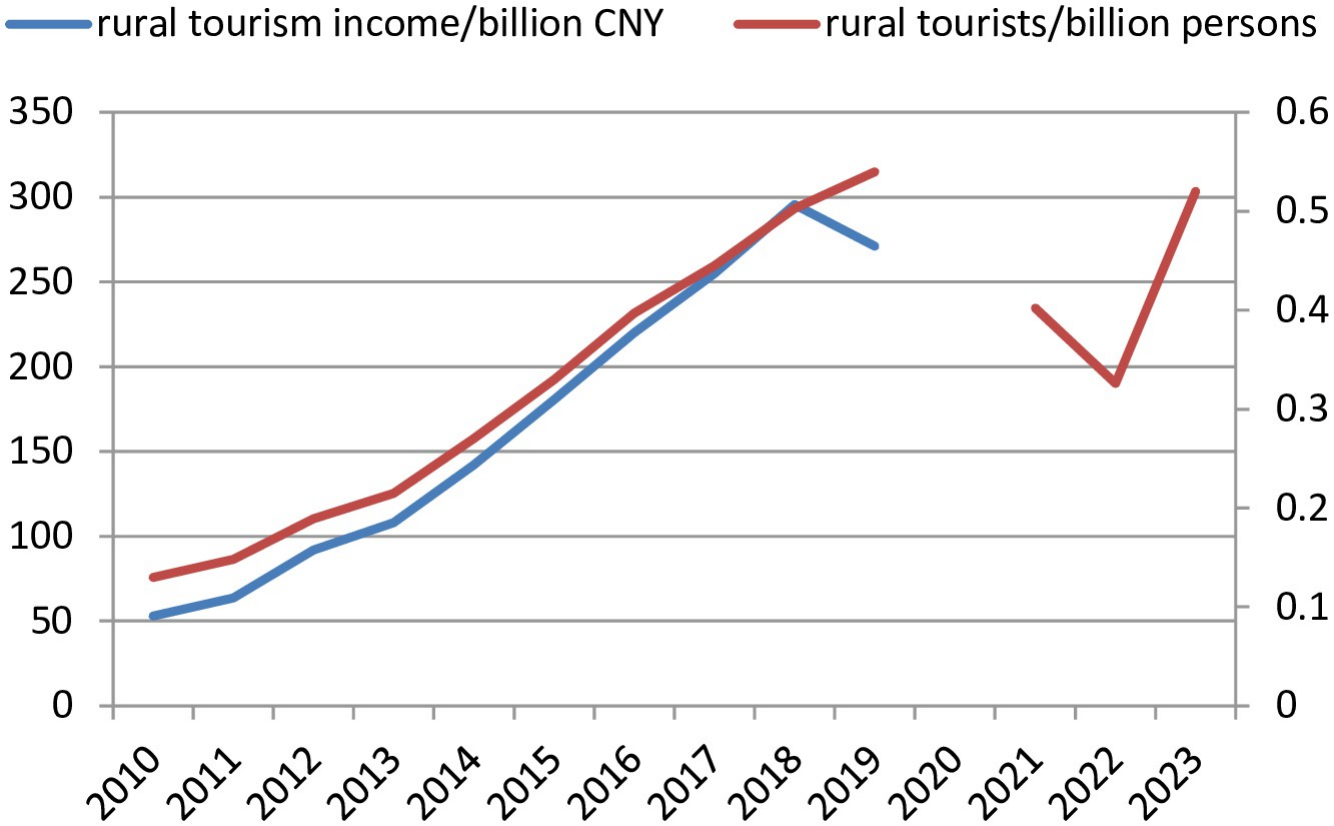
Rural tourism rapid development in Shandong Province.

Currently, many scholars and policymakers have conducted research and practice on PPP models, but they mainly concentrate on national infrastructure or public services. As far as the combination of PPP models and tourism is concerned, academics tend to select the emerging tourist attractions with large passenger flows and abundant resources (such as famous scenic spots or cultural and creative towns) as case studies, but rarely pay attention to rural tourism projects with low investment and a long payback period, like rural cultural industries, traditional villages, etc. It has unable to gain in-depth understanding of PPP models operation and give scientific guidance in rural tourism. In this context, this study conducts a field survey on rural tourism projects adopted PPP models in Shandong Province, to identify the obstacles and difficulties in the implementation of PPP models during rural tourism development. Statistics show that rural tourism in Shandong Province is experiencing a rapid development during 2010–2023 in Fig 1, with 40.8% and 38.3% growth rate in rural tourists and rural tourism revenue, respectively (Above date sources: Report on the Development of Rural Tourism in China(2022), https://xianxiao.ssap.com. cn/catalog/6434897.html). Since 2023, it has cultivated over 3500 large-scale rural tourism attractions, 54 national key rural tourism villages and towns, and 16 rural homestay clusters with various different PPP models; And, there are a total of 12.1 billion investment and 7.98 billion financing demand in local rural tourism development (Above date sources: http://sd.china.com.cn/a/2023/shouyejinriyaowen_0327/1047152.html). Thus, Shandong Province is a typical market holding prosperous rural tourism demand and investment, and it is significant contribution to learn rural tourism projects PPP models’ operation mechanism and impact factors in China. Especially, to identify the underlying reasons for the bad performance or failure of projects, it tries to clarify their operation mechanisms from the aspects of project input and output, risk and benefit, etc. By identifying the problems with PPP models in the rural tourism projects development of Shandong Province, it not only further enhances its rural tourism core competitiveness, but also facilitates the maturation and publicization of PPP models in promoting rural tourism development in other regions. The findings of this study can worldwidely offer new ideas and directions for the development of rural tourism, and further expand its popularity and reputation in developing countries to resolve the issue of insufficient funding for rural tourism development. In addition, the research results are also equipped with a unique practical significance for driving rural tourism development model shift from a "sole focus on development" to an "equal emphasis on protection and development".

## Literature review

### DEA models

Data envelopment analysis(DEA) is a multivariate efficiency evaluation method proposed by American operations researchers Charnes and Cooper, to evaluate relative efficiencies and efficient boundaries [1]. It is a non-parametric method that evaluates the relative efficiencies of decision-making units(DMUs) (such as enterprises, organizations, or entities) under multiple input and output indicators [2]. The basic principle of DEA is to transform the inputs and outputs of a DMU into points in a multidimensional space and evaluate efficiency by determining the boundaries of these points. In DEA, a DMU will be considered relatively efficient if it is located on the efficient boundary, and relatively inefficient if located within the efficient boundary [3].

One strength of DEA is that it does not have to assume a specific form of efficiency function, for which it can be applied to various types of data and contexts. It is available for evaluating the efficiencies of organizations, such as enterprises, hospitals, and schools, as well as the economic efficiency of a country or region. In DEA, envelope analysis is a common method used to determine efficient boundaries. To be specific, it determines efficient boundaries by searching for points that can fully envelop (or cover) other DMUs. These enveloped DMUs are considered the most efficient, while others on the efficient boundary are deemed to be relatively efficient.

The key to a DEA model lies in evaluating the selection of DMUs and determining input and output indicators. DEA obtains the relative efficiency indicators of each DMU by comprehensively analyzing their input and output data. Then, all DMUs are ranked by efficiency indicators to identify relatively efficient DMUs, providing management decision-making information for managers.

### Super-DEA

Traditional DEA models are widely used and require linear programming to identify points at the frontier as the goal of improving inefficient DMUs [4]. They mainly focus on the relative efficiency of a DMU compared with other units and try to determine the reference set of the optimal units. However, as a result of technological progress and data growth, traditional DEA models now face some limitations in terms of computational efficiency. Traditional DEA models, such as the Banker—Charnes—Cooper model, have not taken into account the impact of unit economies of scale. The term “economies of scale” refers to the phenomenon where the expansion of unit production scale reduces unit costs and improves efficiency. Traditional DEA models neglect the effect of economies of scale, which may cause biases in evaluating unit efficiency. In addition, the fact that traditional DEA models mainly focus on the relative efficiency of a DMU compared with other units, means that the efficiency evaluation results of the unit are based on the existing set of units. This relative evaluation may lead to competition between units and limitations to efficiency improvement. Moreover, it is difficult to simultaneously evaluate the efficiencies of DMUs from different years using traditional DEA models, which partially explains why the DEA Malmquist model analysis has been so popular in recent years [5].

The efficiency values of DMUs under traditional DEA models are within the range of [0,1]. When the input reduction rate of a DMU is θ = 1, the DMU is at the efficient frontier, which implies a perfect technical efficiency; when θ<1, there is an efficiency loss of 1-θ [4]. DMUs at the efficient frontier, on the other hand, all have a technical efficiency of 1, making it difficult for them to be further analyzed or ranked. To address this issue, Anderson et al. introduced the concept of super-DEA [6]. The super-DEA model not only considers the comparison of a unit with units but also takes into account the comparison with its own performance. The efficiency values of DMUs measured by the super-DEA model can exceed 1, meaning that the goal is to find units that perform better than in the past under the same technical level.

### PPP models and comparison

The origin of PPP models can be traced back to the toll road construction plan in 18th century Europe, but their formation and development in the modern sense are mainly attributable to the market-oriented reform of public service supply, based on the introduction of active involvement of the private sector, in the “New Public Management” movement [7]. Raymont first proposed a PPP model for public goods in 1992, which allows government departments and private enterprises to establish cooperative relationships and combines the financing and supervision capacities of the former with the capital operation capability of the latter [8].Graeme et al. [9] conducted in-depth research on Private-Finance-Initiative (PFI), a special PPP model and one of the sources of PPP models. Authors discovered many contradictory issues with existing evaluation models, such as the inapplicability of PPP models in the low-complexity IT industry and the unclear division of duties over PPP projects in many European and American countries. By analyzing the PPP projects in the extension engineering of the Port of Rotterdam in the Netherlands, Van Ham et al. [10] identified the risks and difficulties faced by PPP models. However, they also believed that PPP models differ from general investment outsourcing models, as the former greatly strengthen the interaction between public and private sectors. However, with the continuous improvement of PPP projects, the effect of traditional contract management on realising the value creation objectives of PPP projects is limited [11]. Liu et al. has based on the view of multifunctional contract, joint-contract functions and finds that contract control and flexibility can improve PPP project value creation. While, Jani et al. maintained that the formation and development of PPP models are the result of both market failure and government failure. PPP models can effectively alleviate the serious consequences of market failure and further strengthen the governance capacity of government through government-enterprise joint ventures and cooperations [12]. Delmon et al. conducted an introductory study on the concept and mode of PPP financing, arguing that PPP models are new models proposed with regard to the organizational structure of projects and that they can accelerate the identification and initiation of financing projects and facilitate government-enterprise cooperations to better serve the people [13].

There are many different financing cooperation models downstream of PPP models. The common business models under PPP include Build-Operate-Transfer (BOT), Build-Own-Operate (BOO), and Private-Finance-Initiative (PFI), each with their own characteristics and benefits for project development [14]. But at present, PPP financing mainly involves two phases, namely, project construction and project operations. While, PPP models present different financing process and project operations principle. In this regard, independent extensive research has been conducted in academia to clarify their respective financing modes and risk divisions. Such as, Kukah et al. [15] assessed the risks associated with project construction using the Delphi method and factor analysis and summarized the principles of risk sharing for PPP models during project construction. And the research found that risk bearing capacity declines among PFI, BOO, and BOT. PPP projects require heavy investments during construction. The government invests in PPP projects mainly by allocating funds to private entities for investment, while private entities can obtain financing through equity financing, debt issuance, etc. As can be concluded from the above studies, PPP models face risks of increased construction costs, delayed deadlines, policy adjustments, and so forth in the financing and construction phases. Therefore, a detailed in-depth survey should be conducted before project planning. While, Ismail et al. [16] constructed a detailed key performance indicator (KPI) model for project construction and supervision under PPP models using the KPI method, setting high requirements for the involvement of public and private institutions in PPP projects. Liu et al. [17] analyzed the financing mode of urban infrastructure construction projects during PPP construction and evaluated the mechanism of risk sharing between the government and the private sector under PPP models, pointing out that public and private sectors should bear corresponding risks based on their respective advantages. For PPP projects risk management, Liu believed that PPP models are characterized by rapid growth in quantity and value and can be applied and practiced on a large scale in China after the perfection of laws and regulations on investment and financing [11].

### Application of PPP models in the tourism industry

PPP models present popular application in industrial and public projects construction, based on its advamtages on fincincing and unique effectiveness. Yang et al. [18] pointed out that PPP business models have an extremely wide range of applications in many fields, including public infrastructure construction, urban rail transit, environmental protection, healthcare, and education, as well as finance, laws and regulations, and operations management, except that the complexity of such applications varies across different fields. To trace to the source, Sun [19] investigated the birthplace of PPP models in the UK and expounded their operation and supervision mechanisms there, suggesting that China should accelerate the construction of sound PPP working mechanisms as well as supporting policies and legal institutions.

With tourism rising in China, large captial has been invested in tourism industry and projects construction. PPP models have also used to help destination financing for tourism projects for the construction of tourism infrastructure. While, Heldi [20] explored the determinants of the success of PPP in the management and maintenance of tangible and intangible cultural heritage, and to identify what are the parameters of the main factors of sustainable tourism destinations. Cooperation between municipalities and the private sector in the field of tourism also been studied, Beresecká and Papcunová [21] selected model of PPP in the field of tourism at the local level in the conditions of the Slovak Republic, to learn their relationships and PPP working process. Tavana et al. [22] used a weighted influence non-linear gauge system, and developed an integrated multicriteria decision-making and optimization model for partner selection in PPP projects in cultural heritage renovation and the hospitality industry. The current tourism model based on luxury hotel resorts in the Gulf of Papagayo (Guanacaste, Costa Rica), was largely affecting the living condition of its nearby communities, which aimed to discuss the importance of promoting PPP models as innovative forms of governance to increase the sustainability of tourism [23]. The study of PPP models in Chinese tourism industry is also increasing in the international literature. Such as, Cheng et al. [24] discussed PPP matter to sustainable tourism development from the perspective of the spatial effect of the tourism PPP policy in China, and there are significant spatial disparities in the tourism PPP projects. Hu and Wang [25] used in-depth interview methods, and found that the successful cultural tourism PPP model must focus on industrial development, providing comprehensive, full-service, and paying attention to long-term and efficient operation.

As can be seen from the above literature review, PPP models started relatively late in China but developed rapidly, going through stages of theoretical introduction, experience introduction, preliminary exploration, and in-depth research. So this paper viewed performance analysis of PPP models in rural tourism projects of Shandong Province in China, to enrich research gap.

## Research methods and design

### Sample selection and description

This study has selected 12 rural tourism projects (present in Table 1) related with PPP models currently in operation in Shandong Province as its research samples, from the rural tourism projects management platform attached to Shandong provincial department of cultrue and tourism (http://whhly.shandong.gov.cn/). The selection of samples is based on an extensive and comprehensive consideration of data availability, typicality, and familiarity by field survey, Internet information, expert opinions, and opinions of practitioners in this field.

**Table 1:** Brief information on rural tourism projects related with PPP models in Shandong Province.

Name	No.	Type	PPP Models	Payback Mechanism
Rural Revitalization of Liutuan Town, Changyi—“Silk Town” Culture-tourism Integration and Infrastructure	DMU1	Stock	TOT	VGF
Eco-tourism Exquisite City & Old Residential Community Renovation Supporting Infrastructure Construction in Donggang District, Rizhao	DMU2	New-built	BOT	VGF
Eco-tourism & Infrastructure Construction in Lanshan District, Rizhao	DMU3	New-built	BOT	VGF
Changyi Wenshan Forest Park & Museum	DMU4	New-built	BOT	VGF
Municipal Public Facilities & Exhibition Hall Construction in Qingzhou Ancient City	DMU5	New-built	BOT	VGF
Pingdu Featured Town & Tourism Gateway Construction in Qingdao, Shandong Province	DMU6	New-built	BOT	Government-pays
Shiziliu Area & Yellow River Ancient Village Style Belt Rural Tourism in Binzhou Economic and Technological Development Zone, Shandong Province	DMU7	New-built	BOT	VGF
Water Margin Tourism Center (Phase I) in Yuncheng County, Heze, Shandong Province	DMU8	New-built	BOT	VGF
Maliantai Comprehensive Eco-Environmental Remediation in Linzi District, Zibo, Shandong Province	DMU9	New-built	BOT	Government-pays
Wuyang Lake Tourism Infrastructure Construction in Boshan District, Zibo, Shandong Province	DMU10	Stock + New-built	TOT + BOT	VGF
Dongxi Wetland Tourism Resort Construction in Yuncheng County, Heze, Shandong Province	DMU11	New-built	BOT	VGF
China Peony Garden Project in Mudan District, Heze, Shandong Province	DMU12	New-built	BOO	User-pays

The paper summarizes the general PPP models of selected rural tourism projects, providing references for the construction of rural tourism projects in Shandong Province. The selected samples were all approved during 2016 to 2020. Specifically, four of them involve tourism infrastructure construction, four relate to eco-tourism, and seven are cultural tourism projects. All projects were initiated by the government, each with an investment of over 180 million yuan. Among the approved projects, only two are stock projects, which adopt the models of TOT and TOT + BOT, respectively. All other projects are newly built based on the BOT model, except for the China Peony Garden Project in Mudan District, Heze, Shandong Province (BOO). Under the BOT model, the government has a high equity ratio (usually above 10%). Conversely, under TOT and BOO models, the government invests less or even zero. Thus, the project company alone serves as the financing entity in most cases to raise funds from outside, while the government provides feedback to private enterprises based on a payback mechanism. Usually, the government provides payback to partners in the forms of “Viability Gap Funding (VGF)” and “government-pays”. VGF involves circumstances where user-pays are insufficient to guarantee cost recovery and reasonable payback for social capital or project companies, making it necessary for the government to provide them with economic subsidies in the form of fiscal subsidies, equity investment, preferential loans, or other preferential policies. By contrast, under the payback mechanism of government-pays, the government pays the project company based on the availability, operating costs, and performance appraisal of new-built public products as agreed upon in the contract. The payback mechanism of government-pays is often used under BOT and BOO models.

Based on the theory of DEA, these 12 rural tourism projects are treated as DMUs, and an evaluation indicator system is constructed to calculate their operation efficiencies. Next, a detailed introduction is given to the project goals and operation plans of these 12 DMUs. Brief information on the samples is provided in Table 1.

### Indicators system construction and variables

The goal of PPP models is to complete projects by cooperation of government and private enterprises, and improve social benefits. For the DEA process, it is necessary to construct an evaluation indicator system, selects input and output variables, and ensures a one-to-one correspondence between each DMU and the evaluation indicator system. The selection of indicators must be representative, reflecting the basic characteristics of samples while being able to quantify and compare them. The construction of indicators for DEA must follow four principles, the concrete content referring to literature [26].

Considering the features of rural tourism projects, available panel data, and the operation mechanism of PPP models, this study designs an evaluation indicator system by extensively referring to existing literatures. The evaluation indicator system constructed in this paper is shown in Table 2. In terms of capital investments indicators, two indicators are selected, namely, the total investment and “project capital”; In terms of material inputs, three indicators are adopted, namely, project land area, human capital investment, and tourist carrying capacity; In terms of time investments, the project cooperation period is taken as the evaluation indicator. In terms of output indicators, four indicators are used, namely, total resolved local debts, annual operating income, job opportunities created for society, annual tourist reception capacity, and net profit.

**Table 2:** The evaluation indicator system of input and output used in DEA.

	Indicators	Indicators discription
Input	Total investment	Regardless of their specific models, all rural tourism projects inevitably involve investments from both the government and social capital; The total investment is the sum of “amount of capital contribution by the government” and “amount of social capital investment”.
	Project capital	Project capital is often used in a PPP model to establish a project company jointly operated by the government and an enterprise.
	Project land area	Land is an important project resource, and the land area of a project often represents its scale. Major projects also have detailed planning and layout of land resources.
	Human capital investment	Human capital investment is measured in monetary terms, including both the salaries of projects employees and the expenses of hiring experts or introducing talents.
	Tourist carrying capacity	Tourist carrying capacity is a measure of the scale, management efficiency, sustainable development, and other indicators of tourism projects.
	Cooperation period	The cooperation period of a project, consisting of its construction period and operation period, represents an initial estimate of its scale by the government and the enterprise and the expected intensity of government subsidies.
Output	Job opportunities	Job opportunities created for society belong to the category of improving local social benefits. They not only involve social workers in project construction but also attract talents from universities or other regions to contribute to local economic development.
	Annual tourist reception	Annual tourist reception capacity reflects the scale and operation capability of tourism projects, serving as a common evaluation indicator for such projects.
	Annual income	Annual operating income and net profit, directly reflect the revenue level and investment efficiency of a project operated by PPP models.
	Net profit	

At last, it is worth noting that the indicator system has a total of six input indicators and four output indicators. In DEA, there are certain limitations to the relationship between the number of DMUs under evaluation and the number of indicators, that is, the number of DMUs under evaluation should not be less than twice the number of indicators [27] and not be less than the product of the number of input indicators and the number of output indicators [28]. Thus, the indicators in the indicator system need further screening and measurement. In subsequent sections, the entropy weight method (EWM) will be introduced to rank input and output indicators by importance and select them for DEA calculation.

### Data collection process and indicator system choice based on EWM

To collect the indicator data, the paper has firstly searched for the basic information of rural tourism projects, including approved time, investment scale, land area, tourist carrying capacity, annual tourist reception and PPP models, by the internet and built a database; Secondly, authors make an effort to contact the projects to attain the missing data on the internet, like, project capital, human capital investment, and job opportunities. However, some data involves business secrets, like annual income and net profit; authors only collect them via oral communication with rural tourism project manager under the premise of not publication these sensitive data.

In the construction and appraisal of indicator systems, the interactions between different categories and indicators, their impacts on the results, and the degrees of their importance all vary across different fields. This means that we need to determine the weights of relevant indicators to perform a scientific and objective evaluation based on the impacts of different indicators on the results. Taking into account practical factors as well as the numbers of indicators and DMUs, this paper adopts EWM to determine indicator weights and further screen for indicators. EWM is a multi-criteria decision-making method that is often used to assign weights to indicators or determine the importance of multiple indicators (or factors) in decision-making.

The main steps of EWM are as follows. The first step is data standardization. Due to differences in the units of indicators, there are significant differences in their numerical values. In the absence of standardization, the calculation results are likely to include unscientific and unreasonable efficiency values like 0, which will exert a significant impact on or even cause great discrepancies in the calculation results. The process of data standardization consists of normalization and non-dimensionalization. Since the data indicators used in most studies on DEA efficiency assessment are either positive or negative, linear proportional transformation is often performed to construct decision matrices. In this paper, the data indicators do not involve significant negative outputs or a crossover between positive and negative indicators, but the data involved in DEA include both input and output indicators, i.e., cost and benefit indicators. Therefore, the range transformation method is adopted to standardize the data. The main step of range transformation is to perform non-dimensionalization by transforming the initial decision matrix. The standardization of cost indicators is as follows:

$$Y_{ij}={\left(b_{ij}\right)}_{m*n}=\{\begin{array}{c} b_{ij}=\frac{a_{j}^{max}-a_{ij}}{a_{j}^{max}-a_{j}^{min}}\;is-\\ b_{ij}=\frac{a_{ij}-a_{j}^{min}}{a_{j}^{max}-a_{j}^{min}}\;is+ \end{array}$$ (1)

The second step is to determine the proportion (*P*_*ij*_), i.e., the contribution degree, of each indicator in a PPP project. The process of weight determination relies on the concept of information entropy, as expressed below:

$$P_{ij}=\frac{Y_{ij}}{\sum_{i=1}^{m}Y_{ij}}$$ (2)

Since the data of the 12 rural tourism projects collected in this paper (cross-sectional data) lack time-series variation, the actual meaning of *P*_*ij*_ is the proportion of the *j*th indicator in the *i*th rural tourism project. Then, the information entropy (*E*_*j*_) corresponding to each indicator is calculated. Information entropy is a measure of uncertainty, i.e., the probability of occurrence of discrete stochastic events. Simply put, "the more chaotic the situation, the greater the information entropy; and vice versa” [29]. Information entropy can be calculated by the following formula:

$$E_{j}=-k\sum_{i=1}^{m}P_{ij}lnP_{ij}$$ (3)

Where *k* = 1/*lnm*, a constant used to keep information entropy within the interval of [0,1]. If *P*_*ij*_ is calculated to be 0, *P*_*ij*_ *lnP*_*ij*_ will approach 0 infinitely, in which case further coordinate transformation should be performed [30]. However, this is not the case with the data collected in this paper.

Once again, it is necessary to determine the difference coefficient (*D*_*j*_) between indicators. The difference coefficient can be used to observe the contribution of each indicator to the overall coefficient and to compare their internal consistency. The difference coefficient can be calculated as follows:

$$D_{j}=1-E_{j}$$ (4)

Finally, the weight of each indicator (*W*_*j*_) is calculated according to the difference coefficient (*D*_*j*_). Indicator weights are calculated as follows:

$$W_{j}=\frac{D_{j}}{\sum_{j=1}^{n}D_{j}}$$ (5)

The weight vector of the entire indicator system is *W*_*i*_ = (*W*_1_,*W*_2_,*W*_3_*…W*_*m*_).

According to the above entropy weight calculation method, the weights of all input and output indicators are summarized in Tables 3 and 4, respectively. For the convenience of expression, input indicators *I*_1_–*I*_6_ denote project total investment (10,000 CNY), project land area(m^2^), project capital (10,000 CNY), human capital investment (10,000 CNY), cooperation period (years), and tourist carrying capacity (10,000 CNY), respectively. Output indicators *O*_1_–*O*_4_ denote annual operating income (10,000 CNY), job opportunities created for society, annual tourist reception capacity (10,000 persons), and net profit (10,000 CNY), respectively.

**Table 3:** Weights of input indicators.

	I1	I2	I3	I4	I5	I6
*E*_*j*_	0.905813	0.859236	0.901839	0.881853	0.899551	0.840378
*D*_*j*_	0.094187	0.140764	0.098161	0.118147	0.100449	0.159622
*W*_*j*_	0.13241	0.19789	0.13800	0.16609	0.14121	0.22440

**Table 4:** Weights of output indicators.

	O1	O2	O3	O4
*E*_*j*_	0.876834	0.90702	0.864698	0.913086
*D*_*j*_	0.123166	0.09298	0.135302	0.086914
*W*_*j*_	0.28097	0.21211	0.30865	0.19827

The available indicators are re-examined and screened according to the indicator weights measured by EWM. Due to the small number of DMUs in this paper, the number of selected indicators needs to be reasonable. Finally, three input indicators and three output indicators are selected according to their indicator weights. The number of indicators meets the requirement [31]. The reconstructed indicator system is shown in Table 5.

**Table 5:** The reconstructed indicator system for PPP models in rural tourism projects in Shandong Province.

Input Indicator	Output Indicator
Project land area	Annual tourist reception capacity
Human capital investment	Job opportunities created for society
Tourist carrying capacity	Annual operating income

## DEA indicator calculation results

### Performance analysis based on the standard input-oriented DEA model

The working mechanism of the standard DEA model is as follows. The first step is to derive the efficiencies of DMUs by assuming that there are k DMUs, n input indicators, and m output indicators. Then, the input of each DMU is *X*_*i*_ = (*X*_1*i*_,*X*_2*i*_…*X*_*ni*_)^*T*^ and its output is *Y*_*i*_ = (*Y*_1*i*_,*Y*_2*i*_…*Y*_*mi*_)^*T*^. Some scholars suggest that, when a DMU is a rural tourism project, entropy weight and other weight indicators can be introduced to identify the differences between projects [30]. Thus, input and output weights can be added for the DMU: *V*_*i*_ = (*V*_1*i*_,*V*_2*i*_…*V*_*ni*_)^*T*^ and *U*_*i*_ = (*U*_1*i*_,*U*_2*i*_…*U*_*mi*_)^*T*^. The weights of indicators under DEA models are generated by data-driven mathematical programming without the need to pre-set input or output weights, so they are unaffected by human subjective factors. In this way, the efficiency of each DMU can be calculated:

$$E_{i}=\frac{U^{T}Y_{i}}{V^{T}X_{i}}=\frac{\sum_{p=1}^{n}U_{p}Y_{pi}}{\sum_{q=1}^{m}V_{q}X_{qi}}$$ (6)

On this basis, a linear programming model, or a DEA model in its initial form, can be derived by combining the Charnes-Cooper transformation with the dual programming theory and introducing slack variable *s*^−^, redundant variable *s*^+^, and input reduction rate *θ*, as expressed below [32]:

$$\left\{\begin{array}{c} \text{min}\theta \\ s.t.\sum_{p=1}^{n}\lambda_{i}X_{i}+s^{-}=\theta X_{i}\\ \begin{array}{c} \sum_{q=1}^{n}\lambda_{i}Y_{i}-s^{+}=Y_{i}\\ \lambda_{i}\geq 0,s^{+}\geq 0,s^{-}\geq 0 \end{array} \end{array}\right.$$ (7)

Where *λ*_*i*_ denotes the most efficient DMU, usually located on the efficient boundary. Slack variable *s*^−^ is usually located below the efficient boundary, denoting the gap between the expected output that failed to be achieved and the actual output under the same input conditions. Redundant variable *s*^+^ is usually located above the efficient boundary, denoting the difference between the expected input and the actual input under the same output conditions. Input reduction rate *θ* decides whether a DMU is efficient. When *θ* < 1, the DMU is inefficient and needs to be readjusted. When *θ* = 1, the DMU can be judged to be weakly or strongly efficient (optimal) based on whether slack variable *s*^−^ and redundant variable *s*^+^ are 0. When both are 0, the DMU is strongly efficient; otherwise, the DMU is weakly efficient or inefficient.

Input orientation refers to the minimization of input under a given output [33]. In this paper, the first step is to perform input-oriented ordinary DEA efficiency assessment on 12 rural tourism projects in Shandong Province using the DEA-CCR model of DEAP2.1 software. In input-oriented DEA, the output of the unit under evaluation is considered fixed, while the goal is to minimize the required inputs to maximize its efficiency. Thus, it focuses on how to minimize inputs under given output conditions. This method applies to units that intend to achieve a specific output goal with minimal resources. The results of input-oriented DEA are listed in Table 6.

**Table 6:** Results of input-oriented DEA.

DMU	TE	PTE	SE	RTS
01	0.4667	0.5432	0.8591	Increasing
02	0.3219	1.0000	0.3219	Decreasing
03	0.1739	0.6767	0.2569	Decreasing
04	0.1458	0.2860	0.5098	Increasing
05	1.0000	1.0000	1.0000	Constant
06	0.6921	1.0000	0.6921	Decreasing
07	0.4534	0.4596	0.9865	Increasing
08	1.0000	1.0000	1.0000	Constant
09	1.0000	1.0000	1.0000	Constant
10	1.0000	1.0000	1.0000	Constant
11	1.0000	1.0000	1.0000	Constant
12	0.9603	1.0000	0.9603	Increasing
Mean	0.6845	0.8305	0.7989	

In Table 6, TE denotes technical efficiency, i.e., overall efficiency, PTE stands for pure technical efficiency, SE represents scale efficiency, and RTS is the trend of returns to scale. The assessment results of the standard DEA model are all within the interval of [0,1]. As can be seen from Table 6, three projects are in a state of decreasing scale efficiency, four are in a state of increasing scale efficiency, and the remaining five are all at the efficient frontier. From the perspective of overall efficiency, DMU04 has the lowest overall efficiency (only 0.14), while the average overall efficiency of the 12 projects in rural tourism is 0.6845, suggesting that PPP models in rural tourism projects of Shandong Province have a moderate overall efficiency. Apparently, there is still a certain redundancy, i.e., a certain gap from the optimal input. Regarding pure technical efficiency, all projects in rural tourism of Shandong Province have a high pure technical efficiency with an average of as high as 0.8305. Notably, DMU04 has the lowest pure technical efficiency. This suggests that the management and technologies of these projects are relatively advanced and that the overall input redundancy level is low, meaning that their inputs have been effectively utilized. The overall scale of projects in rural tourism of Shandong Province is also high, with the lowest scale seen in DMU03. The average overall scale efficiency reaches as high as 0.7989, indicating that the investment scale matches the project volume.

To more clearly identify the problems with these projects in terms of resource input and output, this paper introduces Tables 7 and 8, which list the input redundancy and output deficiency of each project under input orientation. To be specific, input redundancy is the absolute value of the algebraic sum of slack redundancy and proportionate redundancy; Expectation is the optimal input and output of a project in the optimal state. The difference between the original input and the expected input is input redundancy; the difference between the original output and the expected output is output deficiency. I1–I3 and O1–O3 in the table denote the three major input indicators (project land area, human capital investment, and tourist carrying capacity) and the three major output indicators (annual operating income, job opportunities created for society, and annual tourist reception capacity) in the newly-established indicator system, respectively.

**Table 7:** Input redundancy of PPP models in rural tourism projects of Shandong Province (input-oriented).

DMU	I1 Input Redundancy	I1 Expectation	I2 Investment Redundancy	I2 Expectation	I3 Investment Redundancy	I3 Expectation
01	-233,263.73	277,403.27	-1,233.31	1,466.69	-45.68	54.32
02	0.00	7,600,000.00	0.00	30,000.00	0.00	1,200.00
03	-1,711,172.14	3,582,142.86	-7,892.86	15,107.14	-389.29	610.71
04	-406,989.26	163,010.74	-4,831.25	1,048.75	-85.68	34.32
05	0.00	100,000.00	0.00	2,200.00	0.00	100.00
06	0.00	200,000.00	0.00	4,000.00	0.00	160.00
07	-10,480,801.71	175,198.29	-1,939.87	1,650.13	-54.04	45.96
08	0.00	76,133.00	0.00	429.00	0.00	100.00
09	0.00	1,355,0000.00	0.00	15,000.00	0.00	2.00
10	0.00	200,100.00	0.00	1,200.00	0.00	5.00
11	0.00	5,830,000.00	0.00	12,140.00	0.00	13.00
12	0.00	800,000.00	0.00	750.00	0.00	10.00

**Table 8:** Output deficiency of PPP models in rural tourism projects of Shandong Province (input-oriented).

DMU	O1 Output Deficiency	O1 Expectation	O2 Output Deficiency	O2 Expectation	O3 Output Deficiency	O3 Expectation
01	0.00	28,000.00	244.12	444.12	248.97	278.97
02	0.00	72,300.00	0.00	1,000.00	0.00	2,329.00
03	3,010.71	65,710.71	0.00	850.00	357.75	1,402.75
04	0.00	10,100.00	179.32	329.32	53.02	83.02
05	0.00	60,000.00	0.00	720.00	0.00	600.00
06	0.00	4,300.00	0.00	500.00	0.00	665.00
07	0.00	26,000.00	24.39	424.39	134.43	264.43
08	0.00	27,000.00	0.00	600.00	0.00	180.00
09	0.00	100.00	0.00	300.00	0.00	5.00
10	0.00	220.00	0.00	200.00	0.00	10.00
11	0.00	1,523.00	0.00	400.00	0.00	193.12
12	0.00	681.00	0.00	153.00	0.00	25.80

### Performance analysis based on the Super-DEA model

Similar to traditional DEA model, the super-DEA model chooses specific DMUs to form the productive efficient frontier (line) based on the selected input and output indicators. Based on traditional DEA models, the super-DEA model re-evaluates each DMU at the efficient frontier (line), that is, excluding existing DMUs and constructing a new efficient frontier (line). However, it is worth noting that the type of frontier line constructed by excluding a DMU under evaluation is meaningless in the overall efficiency evaluation. It is only a tool constructed by the super-DEA model to further evaluate DMUs that meet efficiency standards. Thus, the linear expression for the super-DEA model can be derived:

min[θ-ε(∑i=1nsi+∑i=1msj+)]s.t.∑p=1,i=1nλiXip+si=θXip∑q=1,j=1nλjYjq-sj+=Yjqi=1,2,3…,j=1,2,3…,s+≥0,s-≥0 (8)

Where, ε denotes the Archimedes infinitely small quantity. By transforming DMUs at the efficient frontier, the super-DEA model makes it possible to further measure DMUs with perfect efficiency. Therefore, in addition to standard DEA, super-DEA will also be adopted in this paper to further assess the data results. Super-DEA can be calculated by the following formula:

minθs.t.∑j=1j≠knλjxij≤θxik∑j=1j≠knλjyrj≥yrkλ≥0i=1,2…m;j=1,2…n;r=1,2,3… (9)

It can be seen that the difference between the super-DEA model and ordinary DEA models lies in *j ≠ k*, which means that, when evaluating the *k*th DMU, it is necessary to first exclude the DMU itself and then refer to the efficient frontier composed of other DMUs for efficiency assessment. In this case, the DMU may be located outside the efficient frontier, that is, having an efficiency of greater than 1 in a certain aspect. In this paper, a super-DEA matrix model composed of technical efficiency (TE), pure technical efficiency (PTE), and scale efficiency (SE), is successfully constructed using DEAP2.1 software. The calculation results are presented in Table 9.

**Table 9:** Results of super-DEA.

DMU	TE	PTE	SE	RTS
01	0.47	0.47	1.00	Constant
02	0.32	2.23	0.14	Decreasing
03	0.17	0.93	0.19	Decreasing
04	0.15	0.21	0.70	Decreasing
05	2.67	2.68	1.00	Increasing
06	0.69	1.07	0.65	Decreasing
07	0.45	0.56	0.82	Decreasing
08	4.27	1.28	0.03	Increasing
09	3.75	1.64	2.29	Increasing
10	3.57	1.48	2.41	Increasing
11	2.53	2.95	0.86	Increasing
12	0.96	1.44	0.67	Increasing
Mean	1.67	1.41	0.90	-

According to the calculation results, the average technical efficiency of the 12 projects reaches as high as 1.67. Among them, the DMUs 05, 08, 09, 10, and 11 are all located outside the efficient frontier, indicating that they have effectively utilized the invested resources and technologies. These five projects are distributed in three cities, namely, Changyi, Heze, and Zibo. It should be noted that Zibo owns two projects among these five projects, suggesting that Zibo is ahead of other cities in terms of resource utilization and project development efficiency. From the perspective of pure technical efficiency, the average pure technical efficiency of the 12 projects is 1.41, which is also very high. To be specific, the pure technical efficiencies of the DMUs 02, 05, 06, 08, 09, 10, 11, and 12 are all greater than 1, accounting for more than half of all projects. This suggests that Shandong Province has rich technical support and talent reserves for PPP models in rural tourism projects and that these resources can be effectively utilized. The average overall scale efficiency of PPP models in rural tourism projects of Shandong Province is 0.9, which is high but does not exceed 1, indicating that the vast majority of projects have not achieved the most efficient scale utilization and still have a lot of room for improvement. For the super-DEA model, it is not necessary to measure input redundancy or output deficiency, so the analysis of redundancy and deficiency is performed using the standard DEA model.

### Boston matrix

**Fig 2:**
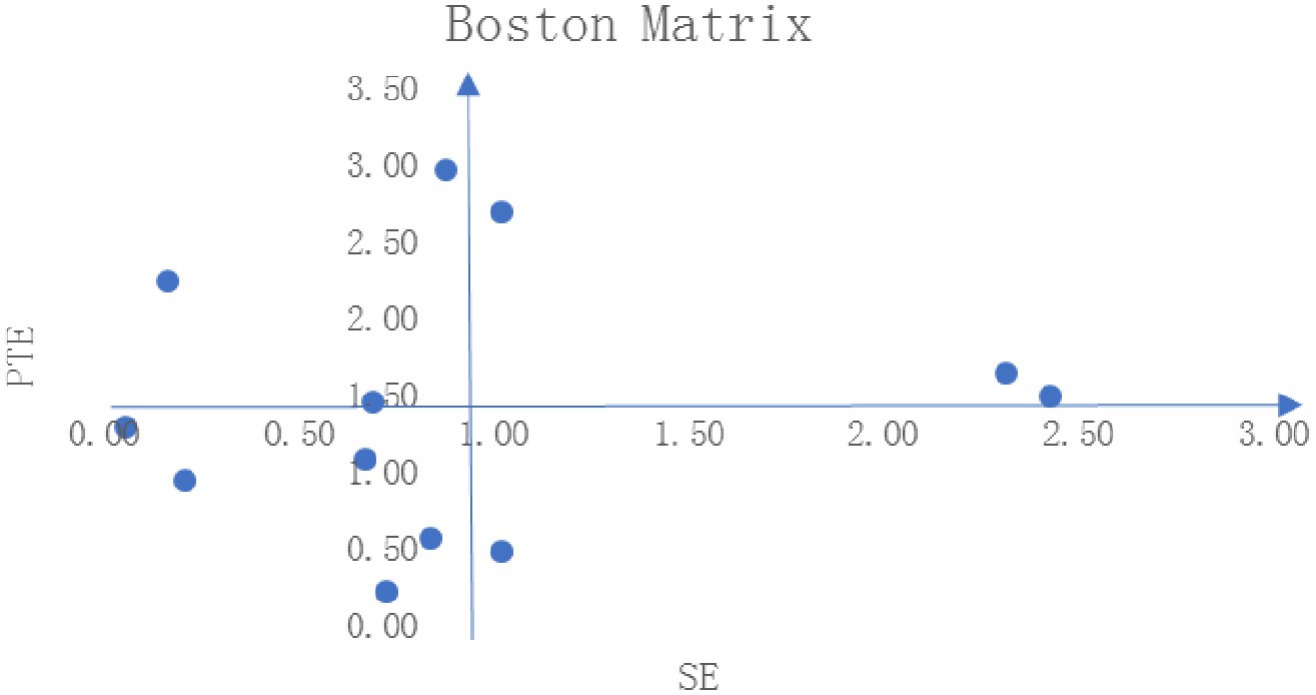
Four-quadrant matrix of pure technical efficiency and scale efficiency.

By introducing the four-quadrant matrix method, this paper evaluates the 12 PPP models in rural tourism projects of Shandong Province from a holistic perspective. The Boston Matrix is one of the most common methods for enterprise and market analysis and the earliest portfolio analysis method. Also known as the Boston Consulting Group Matrix or the BCG Matrix, it is a management tool used to analyze corporate business portfolios. This matrix is typically used to evaluate the relative performance and potential of different products or business units to determine how to allocate resources and investments. In this paper, the average pure technical efficiency and average scale efficiency of the 12 projects are adopted as the y-axis and x-axis, respectively. The completed Boston matrix is shown in Fig 2. The first quadrant contains “Star” projects with high pure technical efficiency and high scale efficiency. The second quadrant involves “Question Mark” projects with high pure technical efficiency but medium to low scale efficiency. “Dog” projects with medium to low pure technical efficiency and scale efficiency can be found in the third quadrant. “Cash Cow” projects with high scale efficiency but medium to low pure technical efficiency are in the fourth quadrant.

In the Boston matrix presented in Fig 2, there are three “Star” projects in the first quadrant, indicating that three PPP projects in rural tourism of Shandong Province have performed well in both scale efficiency and pure technical efficiency. They are DMU05, DMU09, and DMU10. This means that they have strong competitiveness in the market and promising potential for further growth. Therefore, the government needs to consider further allocation of resources to support these three projects, including increasing marketing, R&D, production, and sales resources to further increase their market share and profitability. The second quadrant contains three “Question Mark” projects, namely, DMU02, DMU11, and DMU12. These projects have performed well technically but are less efficient or smaller in scale. It should further standardize their management and operations, and improve their scale efficiency and pure technical efficiency to turn them into a new batch of “Star” projects as soon as possible. There is only one “Cash Cow” project in the fourth quadrant, i.e. DMU01. These three projects enjoy significant technological advantages and are worthy of preservation and further development. Government agencies and operation departments should consider how to further optimize their technologies and improve their pure technical efficiency. The vast majority of projects, as many as five, are “Dog” projects that fall into the third quadrant. Although these projects have performed well technically, their scale efficiency is low. In this case, efforts should be made to improve their overall efficiency by expanding their scale (such as increasing market share, sales, or production scale), or to seek new market opportunities. In addition, it is also worth considering whether to increase resource input to improve their scale efficiency. This may include increasing funding, human resources, or technical support to ensure that these projects operate more efficiently.

## Conclusions and discussions

### Conclusions

This paper has analyzed their respective advantages and disadvantages of PPP models in rural tourism projects of Shandong Province in China. On the one hand, based on the analysis of DEA, super-DEA and Boston Matrix models, there are respectively, three projects with high pure technical efficiency and high scale efficiency, three projects with high pure technical efficiency but medium to low scale efficiency, five projects with medium to low pure technical efficiency and scale efficiency, while only one project with high scale efficiency but medium to low pure technical efficiency.

On the other hand, according to actual assessment results, the overall efficiency of TOT and BOO projects exceeds that of the vast majority of BOT projects, but projects based on other models all have a low scale efficiency, and their construction and financing may rely more on the private sector. However, after the contract expires, the government may need to pay for the transfer of management power. Under the TOT model, the private sector is only responsible for operations, while the risks during the construction phase are largely borne by the government. Moreover, the private sector may not be involved in decision-making on project technologies during the operation phase, which may limit the introduction of technology and management innovations. The BOO model also has the aforementioned defects, and the private sector may continue to have an impact on assets after the contract expires, limiting the flexibility of the project. Overall, the BOT model with higher economies of scale and lower government burdens is currently the best PPP model for the construction of rural tourism projects in Shandong Province. Finally, a comprehensive strategy should be introduced to improve both scale efficiency and technical efficiency.

### Discussions and future work

PPP models present various efficiency performance in rural tourism, while each projects has different financing environment because the various governance, economic policy, private development and tourism market [24]. For PPP models, government supervision and financial support are indispensable and critical factors affecting the efficiency of a project [31]. According to the results of empirical analysis and the field survey, a major reason why many projects have failed to meet efficiency standards or achieve expected goals, lies in the lack of government supervision, while the chaotic internal management of enterprises and the complexity of projects have resulted in slowing project progress. In this context, the government needs to clarify its role and responsibilities in PPP models of rural tourism projects, ensuring that it is both a project supervisor and an active project participant [34].

Comparing international operation methods and rules, common governance and corresponding supervisory between government and private capital, are popular approaches to provide support for efficiency of PPP models [35]. In practice, government from a worldwide perspective, can formulate laws and regulations as well as policies to stipulate its supervisory functions, and the measures that can be taken to assist in the development of PPP models [36]. Specific measures can include improving the transparency of government decision-making and behavior among BOT, BOO, TOT and so on, and ensuring their openness and traceability [24]. At the same time, a sound accountability system should be established to ensure that government officials properly fulfill their project management and supervision responsibilities; effective monitoring and evaluation mechanisms should be introduced to regularly evaluate the impact [30], effectiveness [30], residents and tourists well-being [37] and compliance of projects so that corrective measures can be taken without delay when necessary.

According to the DEA results, projects with low technical efficiency and pure technical efficiency are all large projects with complexity. In a large project, there are simply too many things to take care of, making overall coordination difficult. Existing PPP models of asset projects are mostly government-pays projects, with long repayment periods and high financial pressure. The assets are real estate investments, mostly self-owned leases or operations, with low cash flow, and they have not been effectively revitalized [38]. In the future construction and development of rural tourism projects, the government should consider establishing more cooperation with existing stock projects to reduce construction and development costs and difficulties, based on the international experience of BOT, BOO, TOT and other PPP models. For a large project, thorough market research should be conducted before project initiation to identify tourist demands and market opportunities. On this basis, project scale and position can be well defined to ensure that it does not exceed local resources while meeting relevant demands.

Stock projects are also presenting good performance in terms of overall efficiency and input-output rationality. Replacing new construction with stock projects offers a sustainable approach, as it makes it possible to more effectively utilize existing resources, revitalize stock assets, reduce costs and environmental impact, and improve project sustainability. Introducing more PPP models into rural tourism projects is becoming a powerful way to increase market vitality and combating monopolies. In particular, for large projects with large volumes, long repayment periods, and strong operability, the PPP model of stock project + BOT can better balance risk and revenue [39]. In the phase of project preparation and application, the government should conduct a detailed resource evaluation to identify available stock resources, including land, buildings, and infrastructure. However, the application and implementation of stock projects should be analyzed on a concrete basis case-by-case.

At last, this paper has only focused on PPP models in rural tourism projects of Shandong Province, not paid due consideration to other projects closely related to the tourism supply chain in other fields or public-led, private-led PPP model projects. At the same time, given difficulty of data collection, the indicator system in this paper is relatively simple, and it has mainly discussed government supervision roles in promoting PPP models efficiency, lacking a wide evaluation and exploration. We believe that with further research in other studies and data accumulation, these deficiencies can all be improved in future.

## Supporting information

S1 Data (XLSX)
